# Ethical Issues in HIV-related Social Network Research Involving Substance-Using Sexual Minorities at Risk for HIV

**DOI:** 10.21307/connections-2019.042

**Published:** 2024-08-27

**Authors:** Britt Skaathun, Alera Dermody, Karla D. Wagner

**Affiliations:** 1 Department of Medicine, Divison of Infectious Diseases and Global Public Health, University of California San Diego, La Jolla, CA, USA.; 2 School of Public Health, University of Nevada, Reno, Nevada, USA

**Keywords:** Research ethics, Social network analysis, HIV

## Abstract

**Background:**

Some social network research (SNR) relies on individuals reporting information about network members, with network members not providing consent. We assess how substance-using sexual minorities at risk for HIV perceive the benefits and risks of SNR and the preferred processes for obtaining informed consent.

**Methods:**

We conducted 20 qualitative interviews with adults who identified as people of color, were cisgender male and had sex with cisgender men, and reported using substances (<12 months) in San Diego, CA, USA. Participants were asked about perceived risks and benefits of SNR related to HIV, with differing levels of network information being collected. Participants compared the risks of SNR to risks in daily life and were asked about their preferred consent format. Interviews were recorded via zoom, transcribed, and analyzed using qualitative thematic analysis.

**Results:**

Participants were Latinx (84%), Black (10%), and 1 Filipino (5%), the median age was 31 years, and 25% of them reported previous research experience. Most viewed SNR favorably and less risky than daily life. Participants preferred study designs where network members are also recruited, as their consent was viewed as “community consent.” Participants also felt that community benefits of HIV-related SNR research outweigh the risks. Opinions were mixed about providing identifying information in the context of reporting substance use. A combination of a video using “lay-language” visuals and a written consent format was preferred.

**Conclusion:**

Perceived benefits of SNR to HIV prevention and care outweighed the risks, with concerns about providing last names. Researchers should assess whether the collection of last names is warranted.

## Introduction

The benefits and risks of participating in social network research (SNR) from an ethical standpoint are not straightforward. Ethical research, from a community-based standpoint, focuses on the principles of equity and justice through the sharing of power between the community and academia in response to historic research abuse on marginalized communities (Wallerstein et al., 2019). Equity and justice are sustained through trust, which is a result, in part, of transparency about the research activities and informed choice (Lucero & Wallerstein). SNR is a methodology used to explain the behavior of individuals within a network and of the system as a whole by focusing on specific features of the relationships or interconnections among individuals (Laumann, 1979). Many SNR designs rely on individuals reporting names and information about people in their networks. However, the network members are usually not asked to consent to their information being shared, which poses an ethical dilemma regarding the extent to which network members have truly informed choices about their participation. The goal of this study is to assess how members of a community of sexual and gender minorities of color, a group heavily impacted by HIV, perceive the benefits and risks associated with HIV-related SNR and how they formulate behavioral and attitudinal norms regarding SNR in the context of HIV and substance use research. Findings from this study will help inform the consent process with the goal of reducing potential harms to vulnerable populations.

Social network analysis can be organized into two general analytic approaches: ego-centric (examination of the ties, attributes, and local structure in one’s personal network) and sociocentric (examination of “whole” network structural characteristics for a population). Most SNR in populations affected by HIV/sexually transmitted infections (STI)s, where the underlying population is difficult to enumerate (such as people who use drugs and sexual and gender minorities), typically relies on egocentric social network data, as it is not possible to obtain a roster of individuals in these populations required by the sociocentric network approaches. In egocentric studies, it is assumed that information participants provide about their network will result in network fragments of unique, unconnected egocentric networks within a community. Outside of respondent-driven sampling and studies that include only a very small community, the various participants may not know each other.

One goal of SNR is to explain the behavior of individuals within a network by focusing on specific features of the relationships or interconnections among individuals (Laumann, 1979). Relationships influence a person’s behavior beyond their individual attributes (Blau, 1994). Thus, relationships or linkages within a social setting play a critical role in shaping the spread of health behaviors and attitudes. In the context of HIV, the primary reasons networks are important to examine are: (1) they impact viral transmission directly via patterns of sexual contact; (2) they impact the spread of information (e.g., about the availability of HIV prevention resources); and (3) peer influence impacts the formulation of norms (e.g., drug use and condomless sex practices). All these factors are both risk factors for HIV acquisition and avenues for HIV prevention/intervention. Assessing networks in the context of HIV compounds the ethical considerations of SNR as participants are often asked to disclose illegal or stigmatizing information about network members (Woodhouse et al., 1995).

As with all human subjects research, the application of SNR requires that the methods are ethical, as outlined in the Belmont Report (“The Belmont Report. Ethical principles and guidelines for the protection of human subjects of research”). The Belmont Report outlines three basic ethical principles that should be met: (1) respect for persons (i.e., people should be treated as autonomous), (2) beneficence (i.e., maximize benefits and minimize harms), and (3) justice (i.e., the fair selection of research subjects) (“The Belmont Report. Ethical principles and guidelines for the protection of human subjects of research”). However, in the context of SNR, determining whether these principles are met is challenging due to the nature of the research design. There are varying levels of ethical challenges that arise with social network methodology. Some SNR designs require names and sensitive information (such as drug use and sexual behaviors) of network members to be shared by the primary participant without their consent, and in some circumstances, these names are linked across participant networks, providing multiple accounts of the secondary subjects’ personal information. Even without names being provided, unique information about participants could reveal their identity in a network. These network members are considered *secondary subjects* of the research by many Institutional Review Boards (IRB)s because information about them is being shared, yet it is very difficult to obtain their consent, as recruiting and enrolling every person named in a social network survey is challenging. Therefore, the respect for persons principle (that human subjects must be treated as autonomous and able to make responsible choices) is challenged due to secondary subjects not having the opportunity to consent to information about them being shared. Federal regulations (45 CFR 46.116(d)) state, however, that a waiver of consent can be approved if the following conditions are met (University of California, 2021):

1. the research presents no risk of harm to the secondary subjects, considering probability and magnitude, greater than those ordinarily encountered in daily life or during routine examinations or tests;
2. the research could not practicably be carried out without the requested waiver;
3. if the research involves using identifiable private information or identifiable biospecimens, the research could not practicably be carried out without using such information or biospecimens in an identifiable format; and
4. the waiver will not adversely affect the rights and welfare of the secondary subjects.

Therefore, many IRBs grant such a waiver in the case of SNR research. However, the complexity and potential risks associated with this research design are challenging to portray during the consent process. At the same time, transparency is particularly important in reducing the power hierarchies perpetuated within research and increasing trust among groups who have been historically oppressed.

The issue of secondary subjects has been raised previously, noting that although investigators who administer SN inventories are not interacting with named network members, they are often collecting identifiable information and/or information about alters that is sensitive (Klovdahl, 2005). Klovdahl (2005) recommended a conservative approach that presumes alters named during an SNR are human subjects under the Common Rule, but that this presumption should not bring ethical and beneficial SNR to a standstill. If SNR can be ethically designed and implemented without obtaining informed consent from network members, it should proceed (Klovdahl, 2005). Borgatti and Molina (2005) highlight key points to improve informed consent and the ethics of network research. They suggest two “critical items”: (1) a disclosure contract indicating how network data will be used and who has access to it and (2) a “truly informed” consent form, where participants are shown the disclosure contract and are informed about the specific outputs planned for the study (Borgatti and Molina, 2005). Other “desirable items” include: (1) anonymization and aggregation of results and (2) ensuring that participation is uncoerced. More recent literature has focused on examining privacy concerns related to providing information on network members among sexual minority men (SMM) and transgender women (TW) and suggests involving members of the study population in the design of the study and data collection. (Rudolph and Young, 2019).

The goal of this manuscript is to expand upon this conversation by providing insight into how to improve the SNR consent process with input from a community impacted by HIV in the United States on multiple aspects of SNR design. Currently, there are few guidelines around ensuring that the benefits and risks of HIV-related SNR are being comprehended by primary participants, or what confidentiality protections are needed to best protect secondary participants. Further, affected community members are rarely engaged in developing the consent process. The objective of this study is twofold. The first objective is to assess how sexual minorities of color who use drugs conceptualize the benefits and risks to themselves and others (including network members and community) associated with three typical SNR designs in relation to traditional epidemiologic survey designs where participants only provide information about themselves: (1) egocentric designs in which ego is asked to report about network members, (2) egocentric designs in which network members are also recruited into the study, and (3) egocentric designs in which methods are used to link network members described in multiple participant networks. The second objective is to determine what information and what format the community would prefer a consent form to be in for this type of research. To our knowledge, this study is the first to assess community perspectives on different aspects of SNR design, providing more comprehensive considerations about how to reduce potential harms among vulnerable populations.

## Methods

### Setting and Recruitment

The study took place in San Diego, CA, USA, which is an Ending the HIV Epidemic priority jurisdiction (Fauci et al., 2019). The focus of the present study was on sexual and gender minorities of color both because they are heavily impacted by HIV and because they experience stigma and distrust from/of the medical community, which could further exacerbate mistrust in biomedical research on HIV and substance use (Ogunbajo et al., 2021; Quinn et al., 2017; Remien et al., 2015). In 2019, new HIV diagnoses in San Diego, CA, USA were predominantly among males (90.1%; 78% of whom are SMM and people of color (65%)). In 2019, 34% (1,055) of patients at the sexual health clinic, Good to Go (G2G), reported ever using recreational substances in their lifetime, with 18% (578) reporting use in the last 3 months. Thus, addressing ethical concerns of research participation among SMM who use substances is important in the context of HIV research. A community advisory board (CAB) was involved with the study design and interpretation of the results. Specifically, the CAB played a crucial role in ensuring participants understood the complex design of SNR, including an explanatory video described below.

Recruitment occurred between April 21, 2022 and January 31, 2023. Participants were recruited from G2G (which predominantly serves sexual minorities), peer-referral, and through the help of the CAB (i.e., through the identification of community members fitting the criteria). We employed several strategies to address recruitment strategies, including repeated follow-up contacts, flexible scheduling to accommodate participants’ availability, and emphasizing the significance of the research to build trust and highlight its potential benefits to the community. Social media platforms were also used to broaden recruitment efforts. Posts were made asking community members to refer individuals who fit the study criteria and were willing to participate in an hour-long remote interview. This strategy leveraged the social networks of community members to reach a wider and potentially more diverse pool of participants.

The Institutional Review Board at the University of California, San Diego approved all study procedures.

### Data Collection

Participants were eligible if they met the following criteria: (1) age 18 years or older, (2) identify as Black and/or Latinx; (3) assigned male sex at birth and have sex with cisgender men or identify as non-heterosexual; (4) speak English; (5) reside in San Diego County; and (6) report substance use in the past year (defined as any illegal substance).

Two interviewers conducted one-on-one semi-structured qualitative interviews that elicited participants’ perspectives on the risks and benefits of participating in HIV-related SNR studies, as well as their perspectives on how they would prefer the informed consent information be presented. We collected demographic variables including age in years, gender identity, ethnicity, race, and the type of substances used in the past year. The interview began by asking participants questions about their research experience and their thoughts about the benefits and risks of participating in HIV research, in general (i.e., traditional epidemiology designs). For instance, the interview began with the following, “I want to start with some bigger picture questions to learn about your experiences. Please feel free to give as much detail as you feel comfortable with, but also remember that if you’re not comfortable talking about something, you can just let me know and we can move on. Have you ever participated or known anyone else who has participated in a research study? What was it about? How did that experience go for you?”

Given the abstract nature of the study topic and participants’ limited familiarity, an animated three-minute video was shown that describes SNR methods, the types of data collected (i.e., sensitive information about network members, such as drug use), how the data could be used (including restrictions on data use, such as researchers never contacting alters), and potential benefits and risks of SNR prior to asking questions about potential participation in SNR (https://youtu.be/Y8kXKz5vVVU). The video was introduced by stating the following, “Now I’m going to shift the focus a little bit and show you a short video that describes a study design that we call ‘SNR’ In some ways it’s like a traditional public health study, but there are some unique aspects. After you watch the video, I’m going to ask you some more questions about what you perceive to be the positives and negatives of this type of study.”

Participants were then asked about the themes related to the perceived risks and benefits to themselves, others, and the community with regard to common elements of SNR including: (1) egocentric designs in which the participant is asked to report about network members, (2) egocentric designs in which the alter reports about the participant, (3) egocentric designs in which network members are also recruited into the study (i.e., peer-referral of a sample of their network), and (4) egocentric designs in which entity resolution procedures are used to link network members described in multiple egonets. Following these questions, we asked participants to think about two hypothetical scenarios to assess whether they perceived the risks of participation in HIV-related SNR to be, “greater than those [risks] ordinarily encountered in daily life.” One scenario in which they are talking about friends and/or sex partners in a public space (i.e., bar/café) and the conversation is overheard, and another scenario in which personal information about them may have accidentally shared against their will via their phone being lost and broken into. Participants were then asked to comment on the perceived risk of this scenario versus the perceived risk participation in HIV-related SNR. Finally, we asked participants about their preferred consent format. Interviews were conducted and recorded via Zoom (audio only). Participants were offered an incentive of $50 in the form of an Amazon gift card for participating in the interview.

### Analysis

Audio recordings were transcribed verbatim and transcripts were loaded into ATLAS.ti version 22.2.3 (Scientific Software Development, 2022) for organization and coding. Two authors read the transcripts independently in their entirety and developed a series of open codes and memos to characterize emergent themes and document initial impressions (Miles & Huberman, 1994). We organized these codes into a codebook that we then applied to the entire set of transcripts. A total of 31 codes were developed. Memos were further developed to describe the content and relationships among the themes. After an initial round of coding by two authors, the coded transcripts were discussed and codes were further refined. Disagreements were infrequent and discussed with the third author. We output the data from ATLAS.ti and organized the thematically coded data into a set of higher-order conceptual categories corresponding to our research questions. For example, a code of “negative feelings” translated to the theme of “risk” which was related to our research question regarding how sexual minorities of color who use drugs conceptualize the risks of participating in SNR. The thematic analysis was then reviewed with a member checking group (Klinger, 2005). This group consisted of members of the study’s community advisorary board from the same demographic as the study participants who reviewed results and checked them for accuracy. Quotes are provided using a unique respondent identifier.

## Results

A total of 20 participants were interviewed. The participants were predominantly cisgender male (94%), 17 (85%) identified as Latinx, 2 (10%) identified as Black, and 1 (5%) as Filipino, and the median age was 31 years (interquartile range (IQR) 27, 33.5). Participants reported using the following substances in the previous 6 months: Marijuana 15 (75%), cocaine 5 (25%), psilocybin 5 (25%), Methylenedioxymethamphetamine (MDMA) 3 (15%), Lysergic acid diethylamide (LSD) 2 (10%), ketamine 1 (5%), and Adderall 1 (5%). Approximately a quarter of respondents had some previous experience with research. The following section presents results organized by 4 themes: (1) Perceived benefits of HIV research in general and of HIV-related SNR, (2) Perceived risks of HIV research in general and HIV-related SNR, (3) Comparisons between the risks of HIV-related SNR to everyday risks, and (4) Informed consent preferences.

### Perceived Benefits of HIV Research in General and of HIV-related SNR

Participants were asked about the perceived benefits of traditional epidemiologic HIV study designs (i.e., general HIV research from an individual perspective) and HIV-related SNR separately; however, their responses about the two study designs were intertwined. In general, participants felt that HIV research is a way of protecting the lesbian, gay, bisexual, transgender and queer (LGBTQ) community of color through prevention, education, and resource allocation. When asked what he thought about how HIV research might benefit or put the LGBT community at risk, one participant said:

“I would definitely feel that for the LGBTQ community, or the gay community, or the queer community, it [HIV research] would just be positive. I don’t know anybody that is, you know, gay, lesbian, or any of that that is against AIDS research”

-R6

This theme of HIV research benefiting the queer community is further illustrated in the quote below, but pertaining to the public in general (as opposed to the LGBT community specifically):

“Definitely the results of the study, right, are very valuable whether they’re positive or negative. There’s value in any findings of that nature. I don’t feel like research studies have any type of ill or cruel intent behind them. So, if it’s for the greater good and it’s to educate or to empower its findings, then by all means it’s something that I’m all about.”

-R7

We can see how he describes two concepts from the Belmont report and ethical decision-making that are often used to evaluate the overall risks and benefits of human subjects research: beneficence (i.e., maximizing benefits and minimizing harms for participants) and utilitarian reasoning (i.e., considering the greatest balance of benefits over harms for participants and the public/community) (Driver, 2022). Specifically, this respondent highlighted how, regardless of the findings, research that is “for the greater good” is worthy of support.

A few benefits unique to SNR did arise. Many participants likened HIV-related SNR to the public health practice of contact-tracing (identifying and notifying someone that they have been exposed to an infectious disease) that uses network-based methods. They commented on the benefits of specifically identifying transmission links, or “mapping out” transmission that is possible with SNR versus traditional HIV research. For instance, when asked about the benefits of HIV-related SNR, one participant stated:

*“You can trace it [HIV] and kind of stop it*… *Because maybe sometimes people don’t get tested and they don’t really know that they might have been exposed to something. Maybe like that way you can help people or like kind of warn someone and be like ‘Hey, like you might have been exposed. You should go get tested.’ So I feel like that will be a huge benefit to like slow down the spread of certain things. ”*

-R8

The member-checking group highlighted that participants’ understanding of the potential public health impact of the particular study that they are participating in may influence their assessment of whether the benefits of participation outweigh the risks. For instance, the quote by the participant above refers to “contact-tacting” (contacting someone with an infection and asking them to identify people who may have been exposed); however, most SNR studies do not contact members of the networks who were listed by primary participants.

### Perceived Risks of HIV Research in General and SNR

Several concerns arose in relation to both the study designs that pertained to loss of confidentiality. For instance, participants were concerned that if their information were released they would be gossiped about or would feel shame and judgment about the behaviors they reported, and they feared repercussions from their jobs, family, and friends as a result. When asked if they thought there were any risks to participating in HIV research, one participant emphasized shame and judgment. He responded:

“Yeah. I mean, definitely some I’m gonna say a little bit of like guilt shamed for sure…[referring to drug use history]: I know I need to be honest and whatever, but I was like it’s always like so like ‘Oh, God, I have to like answer these questions.’ I’m like ‘Ugh, what am I doing?’”

-R11

However, there were also notable differences in the participants’ assessment of the risks associated with each study design. In terms of risks specific to HIV-related SNR, participants were asked about two categories of perceived risks: risks associated with being talked about by someone in the study (i.e., as a network alter) and risks associated with talking about others. As described above, the SNR design that was explained to respondents included a detailed assessment of sexual and drug use behavior with each alter identified within a network. Regarding concerns about being talked about in the context of the study, those who felt comfortable with their drug use behavior did not mind if they were being discussed by others in an SNR study.

“I personally wouldn’t mind it. I just like know. You know, I know the kind of person I am. Like it’s not like I engage in like recreational drugs like every week or something like that. It’s just like when there’s like—so I know like what I—I guess like my habits or my—I can’t think of the word right now, but like I wouldn’t mind it personally. I would get like why an individual might mind it like if it were someone different. But like if it were me, like it’s fine. I know it’s being used for something good and like that’s the way like my mind works. So like I am happy that they’re sharing my information and that my information is being able to assist like others. Yeah.”

-R14

However, those who feared being judged or misrepresented were apprehensive about being discussed without consent.

“I think it’s the social honestly. Like if some [Laughter]—I mean, just to be frank, but like if someone thinks you’re like a slut, then that’s— that’d be kind of weird, you know. I don’t know.”

-R1

Regarding concerns about talking about others, there was reluctance to provide the last names of network members, out of fear that network members would be contacted by the research team. This highlights the need for informed consent documents and study procedures such as survey instruments and interactions between staff and participants to reiterate a clause stating that information will not be used to contact individuals who participants name. One participant highlights this reluctance by stating:

“like that really identifiable information where you could start piecing them together, at that point, you know, I’m like not gonna give you my friend’s first and last names. At that point, like I know how to find someone and a lot of their history just off their name.”

-R9

Participants were especially concerned about how breaches of confidentiality could put their network members at risk, given the criminalized and stigmatized nature of some of the information they would be asked to report about others:

“I would fear that like their place of employment might found out that they were like engaging with like certain drugs and that like, you know, affecting them like them getting tired or like reprimanded. Let’s say like they were not out of the closet and like their parents found out that they were like doing this and that or their family found out that they were doing this and that, like I would be afraid that it might affect their lives or whatnot.”

-R14

In response to this concern, one respondent suggested that he be offered the opportunity to check with the network members first, before disclosing information:

“Like let me send him a quick text or, you know, just let me get consent first, especially knowing if I have any thoughts about like ‘Oh, how would he think about this?’ Does he even wanna be a part of it? Could it affect his job? You know, what are his thoughts?”

-R 15

Participants were also asked about the perceived risks of using peer-referral as a recruitment method for social network studies, and about the risks of entity resolution (i.e., linking the names provided across participant networks). No concerns were expressed about either topic. Peer-referral was viewed favorably because it was seen as a way of incorporating community consent. That is, participants thought that through a process of referring their network members to the study, the alter’s agreement to participate would signal whether consent to their information being shared was generally acceptable by the participant’s network.

When asked about entity resolution, participants did not view the connecting of individuals from separate egonets into a whole network as posing any greater risk than the disclosure of names in the first place:

“I can still have the same concerns about sharing the names. But that it connects to a bigger like web, no.”

-R6

Another participant described how the connecting of individuals across networks did not change their perception of risk by saying:

”It wouldn’t change them. I do appreciate the reminder of how there’s cohorts of all types of nature around us and sometimes we forget how connected we really are and maybe the missed opportunity sometimes to reach out to someone because of us being intimidated or scared. But in reality, there’s a lot more that we probably know of one another. ”

-R7

### Comparison to Everyday Risks

At the end of the interview, participants were asked to compare the potential risks for participating in HIV-related SNR research to those they might face in everyday life. Participants generally felt that the hypothetical “real life” scenario of gossip being overheard in public was more worrisome than participating in SNR, stating that SNR feels more “safe” because they trust the data security of institution.

“Say if I’m talking to a researcher and they go and disclose something that we agreed not to disclose on, at least I know there’s some sort of repercussion from that versus if I’m the one disclosing it”

The level of trust was higher, and perceived risk was lower, when the hypothetical researchers were described as members of their community, because participants felt this identity makes researchers more likely to have the participants’ best intentions in mind. “Community” was discussed as either people who identify as gay or queer, or people of color. For instance:

“I would be more a lot more comfortable if it’s somebody within the community. And to be more specific, a black indigenous or a person of color.”

-R13

Similarly, the level of perceived risk was higher when trust in the institution conducting the research decreased. One participant described his distrust in the following comment:

“So I’m definitely always gonna be a little skeptical of government doing those type of tests [HIV research] especially specifically on a marginalized community like the LGBTQ and, even more specific, Black LGBTQ.”

### Consent Preferences

Although there was some diversity in opinions regarding the format and delivery of information used in the informed consent process, many preferred a combination of both written and video information. Several participants who identified as “visual learners” stated that they preferred a video that provided a simplified version of the study process. Although many participants stated they seldom read the written consent form, most participants preferred incorporating the written consent as a form of “protection” since it can be referenced in the future if concerns arise. Similar to the findings from the everyday risk-comparison theme, participants seemed to feel more comfortable participating in HIV-related SNR research if there are “consequences” for breached confidentiality. Participants viewed the written consent form as an agreement that leads to “consequences” if broken.

“I feel like if there’s like video component…that can be really helpful to the participant and learning more about what— and just making it super clear what the expectations are, the pros and cons, and the ethics behind it. ”

-R5

“I think the video is extremely helpful because that really just made it clear to me what’s happening and how it’s gonna be used and all that. But having a form definitely makes me feel more safe.

Interviewer Okay.

Interviewee Something that’s a hard copy that I know if something were to go wrong, I can refer back to that and be like ‘Hey, we said this. We signed on this and… What the hell?’ You know?”

-R13

“Like I feel like if there’s like video component like the one that you shared like that can be really helpful to the participant and learning more about what—and just making it super clear what the expectations are, the pros and cons, and the ethics behind it.”

-R 5

## Discussion

This study was motivated by a critical rethinking of what “do no harm” may mean within informed consent practices involved with HIV-related SNR, and understanding what factors influence people’s willingness to participate and how we can reduce potential harms that impact the livelihoods of marginalized communities (Kadushin, 2005). In general, most participants viewed HIV-related SNR favorably and less risky than risks ordinarily encountered in daily life due to confidentiality protections in research. However, the level of trust varied with the type of organization conducting the research, with less trust being placed in large governmental organizations and more trust being placed in researchers who identify with the community being researched. Many participants discussed historic anxieties regarding the United States government, healthcare institutions, and further marginalization from within and outside of their respective communities with regard to sharing information with entities they do not trust. This anxiety and discomfort may lead to compromised accuracy of study data, as a result of participants omitting sensitive information (Kadushin, 2005). These themes speak to the potential of the ethical principles of respect for persons and justice (outlined in the Belmont Report) being challenged when the research is being conducted by non-community members.

In terms of the perceived risks and benefits to themselves, others, and the community with regards to SNR procedures in HIV research, participant opinions were mixed about the level of name data they were willing to provide. Participants were generally comfortable reporting first names, but were uncomfortable reporting last names, particularly in the context of reporting network members’ substance use and sexual behavior out of fear of “outing” their friends or otherwise generating undesirable repercussions from family, friends, employers, or law enforcement. These findings are consistent with other findings from qualitative research with non-Hispanic White SMM, and TW involved in substance use SNR in Kentucky, showing that participants were reluctant to provide last names of their network members because of the feared criminal–legal consequences that could ensue (Rudolph & Young, 2021). These concerns may be mitigated with improved transparency in the consent process, as described below.

The secondary objective of our study was to determine preferences for the types of information and format for presenting informed consent information, and how those preferences would help participants understand the implications and risks associated with disclosing personal information about their network (Borgatti & Molina, 2003). Participants highlighted the complex nature of SNR and felt that the consent process should be simplified to increase comprehension. Specifically, they felt that the use of a video helped with interpretation as most participants did not understand how the name data would be utilized and had trouble comprehending the entity resolution process. Consequently, videos are recommended for study designs that involve an entity resolution process. Participants also perceived the written consent as documentation and “security,” and still wanted to receive it (as is currently required) in addition to the video regardless of the SNR design. Participants expressed concerns about the people they name being contacted by researchers. It is therefore recommended that both the informed consent and the survey clearly state whether or not this will occur. These findings highlight the need to continuously update the consent process and modality to ensure that the benefits and risks of SNR are being comprehended by the study population. For instance, as society becomes more accustomed to receiving information in a digital format, our consent modality should follow as long as they retain all of their required elements. The consent forms in their contemporary text-only forms may present a power imbalance between the community and academia, as academic language may exclude and isolate the community (D’Angelo & Ryan, 2021).

Lastly, one of the most surprising findings from our study was that participants in our study viewed the peer-referral process favorably because they viewed it as an opportunity for network members to consent to their information being shared by primary participants previously. This is counter to the perception of institutional review boards, which expressed concerns of retaliation resulting from peer-referrals realizing their data may have been shared by the primary participant. On the contrary, while the peer-referral process does not actually obtain consent from network members, primary participants felt that the opportunity to recruit was reassuring.

## Limitations

These results represent a small sample of people with very specific identities. The perspectives represented in this study may differ in different geographic locations and by age, gender identity and sexual identity, race, and ethnicity. It should be noted that only a few participants identified as non-Latino, therefore their perspectives may not be generalizable. Similarly, the study was limited to only English-speaking participants, and non-English speaking community members may have different opinions and concerns regarding SNR, especially given their immigration status. It should also be noted that the hypothetical scenario comparison may not be a common daily risk/activity for some people, which could bias their response in either direction depending on the level of everyday risk they experience. It should also be noted that many SNR studies can be done without collection of names of secondary networks. In addition, due to the majority of participants being recruited from the G2G clinic, several of the participants had prior experience with either research, or prior experience with accessing HIV or STI services in an academic setting. This may have resulted in more favorable views about research due to familiarity and trust.

## Conclusion

Participants in this study felt that the benefits of HIV-related SNR to HIV prevention and care outweighed the risks. However, there were concerns about providing last names in conjunction with information about network members that could be damaging, such as substance use. Future research should assess when and whether collection of last names is truly warranted. Previous frameworks have been developed for collecting anonymous network data from sex partners of SMM and could be adapted in circumstances where last names are not necessary (Young et al., 2015). Finally, to increase comprehension and confidence in providing consent, the consent process and modality should keep pace with society, such as by including digital and/or video formats.
